# Novel targeted drugs approved by the NMPA and FDA in 2019

**DOI:** 10.1038/s41392-020-0164-4

**Published:** 2020-05-08

**Authors:** Wenjing Wang, Qiu Sun

**Affiliations:** State Key Laboratory of Biotherapy, Cancer Center, West China Hospital, Sichuan University and Collaborative Innovation Center for Biotherapy, 610041 Chengdu, China

**Keywords:** Target identification, Drug discovery

In 2019, the FDA’s Center for Drug Evaluation and Research (CDER) approved 48 novel drugs [https://www.fda.gov/drugs/new-drugs-fda-cders-new-molecular-entities-and-new-therapeutic-biological-products/novel-drug-approvals-2019],^1^ which was fewer than the all-time record of 62 NTD approvals in 2018,^2^ but it was still a fruitful year. Among these approved drugs, 39 were targeted drugs (Table 1), including 27 small molecules, 3 antibody-drug conjugates (ADCs), 1 RNA interference (RNAi) therapy, 1 antisense oligonucleotide, 4 monoclonal antibodies (mAbs), 1 recombinant fusion protein, and 2 synthetic peptide analogs. The targets included kinases, ion channels, exons, enzymes, and receptors. Oncology, which remains the most important drug discovery area, accounted for 23% (9/39) of the targeted drug approvals.

**Table 1:** Targeted drugs approved by the FDA in 2019

No.	Brand name	Active ingredient	Approval date	Target/Activity	FDA-approved use on approval date*	Drug class	Company
1	Egaten	Triclabendazole	2/13/2019	Microtubule/tubulin	To treat fascioliasis, a parasitic infestation caused by two species of flatworms or trematodes that mainly affect the liver, sometimes referred to as “liver flukes”	Small molecule	Novartis Inc.
2	Zulresso	Brexanolone	3/19/2019	GABA receptor	To treat postpartum depression (PPD) in adult women	Small molecule	Sage Therapeutics
3	Sunosi	Solriamfetol	3/20/2019	Dopamine and norepinephrine transporters	To treat excessive sleepiness in adult patients with narcolepsy or obstructive sleep apnea	Small molecule	Jazz Pharmaceuticals
4	Mayzent	Siponimod	3/26/2019	Sphingosine-1-phosphate (S1P) receptor	To treat adults with relapsing forms of multiple sclerosis	Small molecule	Novartis Inc.
5	Evenity	Romosozumab-aqqg	4/9/2019	Sclerostin	To treat osteoporosis in postmenopausal women at high risk of fracture	Monoclonal antibody (mAb)	Amgen
6	Balversa	Erdafitinib	4/12/2019	FGFR family	To treat adult patients with locally advanced or metastatic bladder cancer	Small molecule	Janssen Biotech
7	Skyrizi	Risankizumab-rzaa	4/23/2019	Interleukin-23 (IL-23)	To treat moderate-to-severe plaque psoriasis in adults who are candidates for systemic therapy or phototherapy	Monoclonal antibody (mAb)	AbbVie Inc.
8	Vyndaqel	Tafamidis meglumine	5/3/2019	Selective transthyretin (TTR) stabilizer	To treat heart disease (cardiomyopathy) caused by transthyretin-mediated amyloidosis (ATTR-CM) in adults	Small molecule	FOLDRX Pharms
9	Piqray	Alpelisib	5/24/2019	PI3Kα inhibitor	To treat breast cancer	Small molecule	Novartis Inc.
10	Polivy	Polatuzumab vedotin-piiq	6/10/2019	Anti-CD79b antibody-drug conjugate (ADC)	To treat adult patients with relapsed or refractory diffuse large B-cell lymphoma	Antibody-drug conjugate (ADC)	Genentech Inc.
11	Vyleesi	Bremelanotide	6/21/2019	Melanocortin 4 receptor agonist	To treat hypoactive sexual desire disorder in premenopausal women	Synthetic peptide analog	AMAG Pharmaceuticals
12	Xpovio	Selinexor	7/3/2019	Selective CRM1 inhibitor	To treat adult patients with relapsed or refractory multiple myeloma (RRMM)	Small molecule	Karyopharm Therapeutics Inc.
13	Recarbrio	Imipenem, cilastatin and relebactam	7/16/2019	Imipenem: β-lactamase inhibitor (previously FDA-approved antibiotic) cilastatin: dehydropeptidase inhibitor (previously FDA-approved antibiotic) relebactam: new β-lactamase inhibitor	To treat complicated urinary tract and intra-abdominal infections	Small molecule	Merck & Co., Inc.
14	Nubeqa	Darolutamide	7/30/2019	Androgen receptor (AR) antagonist	To treat adult patients with nonmetastatic castration-resistant prostate cancer	Small molecule	Bayer Healthcare
15	Turalio	Pexidartinib	8/2/2019	Colony-stimulating factor 1 receptor (CSF1R)	To treat adult patients with symptomatic tenosynovial giant cell tumor	Small Molecule	Daiichi Sankyo Inc.
16	Wakix	Pitolisant	8/14/2019	Histamine H3 receptor inverse agonist	To treat excessive daytime sleepiness (EDS) in adult patients with narcolepsy	Small molecule	Harmony Biosciences
17	Rozlytrek	Entrectinib	8/15/2019	pan-Trk, ROS1, and ALK inhibitor	To treat adult patients with metastatic, ROS1-positive non-small-cell lung cancer (NSCLC)	Small molecule	Genentech Inc.
18	Inrebic	Fedratinib	8/16/2019	JAK2 inhibitor	To treat adult patients with intermediate-2 or high-risk primary or secondary myelofibrosis	Small molecule	Impact Biomedicines, Inc.
19	Rinvoq	Upadacitinib	8/16/2019	Janus kinase 1 (JAK1) inhibitor	To treat adults with moderately to severely active rheumatoid arthritis	Small molecule	AbbVie Inc.
20	Xenleta	Lefamulin	8/19/2019	50S bacterial ribosome	To treat adults with community-acquired bacterial pneumonia	Small molecule	Nabriva Therapeutics
21	Nourianz	Istradefylline	8/27/2019	Adenosine A2A receptor antagonist	To treat adult patients with Parkinson’s disease experiencing “off” episodes	Small molecule	Kyowa Kirin, Inc.
22	Ibsrela	Tenapanor	9/12/2019	Na+/H+ exchanger NHE3 inhibitor	To treat irritable bowel syndrome with constipation in adults	Small molecule	Ardelyx, Inc.
23	Aklief	Trifarotene	10/4/2019	Retinoic acid receptor (RAR) agonist	For the topical treatment of acne vulgaris in patients 9 years of age and older	Small molecule	Galderma R&D
24	Beovu	Brolucizumab-dbll	10/7/2019	Vascular endothelial growth factor (VEGF) inhibitor	To treat wet age-related macular degeneration	Monoclonal antibody (mAb)	Novartis Pharmaceuticals Corporation
25	Scenesse	Afamelanotide	10/8/2019	Melanocortin 1 receptor	To increase pain-free light exposure in adult patients with a history of phototoxic reactions (skin damage) from erythropoietic protoporphyria	Synthetic peptide analog	Clivunel Inc.
26	Reyvow	Lasmiditan	10/11/2019	5-HT1F receptor agonist	For the acute treatment of migraine with or without aura in adults	Small molecule	Eli Lilly
27	Trikafta	Elexacaftor/ivacaftor/tezacaftor	10/21/2019	Elexacaftor: new cystic fibrosis transmembrane conductance regulator (CFTR) modulator ivacaftor: CFTR potentiator tezacaftor: F508del CFTR corrector	To treat patients 12 years of age and older with the most common gene mutation that causes cystic fibrosis	Small molecule	Vertex Pharmaceuticals
28	Reblozyl	Luspatercept-aamt	11/8/2019	Binds several TGF-beta superfamily ligands	For the treatment of anemia in adult patients with beta thalassemia who require regular red blood cell transfusions	Recombinant fusion protein	Celgene Corporation and Acceleron Pharma Inc.
29	Brukinsa	Zanubrutinib	11/14/2019	Bruton tyrosine kinase (BTK) inhibitor	To treat certain patients with mantle cell lymphoma, a form of blood cancer	Small molecule	BeiGene USA Inc.
30	Adakveo	Crizanlizumab-tmca	11/15/2019	P-selectin	To treat patients with painful complications of sickle cell disease	Monoclonal antibody (mAb)	Novartis Inc.
31	Givlaari	Givosiran	11/20/2019	Aminolevulinic acid synthase 1 (ALAS1)	To treat acute hepatic porphyria, a rare blood disorder	Gene therapy: RNA interference (RNAi)	Alnylam Pharmaceuticals
32	Xcopri	Cenobamate	11/21/2019	Sodium channel blocker	To treat partial onset seizures	Small molecule	SK Life Science, Inc.
33	Oxbryta	Voxelotor	11/25/2019	Sickle hemoglobin (HbS) polymerization inhibitor	To treat sickle cell disease	Small molecule	Oxbryta to Global Blood Therapeutics
34	Vyondys 53	Golodirsen	12/12/2019	Exon 53	To treat certain patients with Duchenne muscular dystrophy	Gene therapy: antisense oligonucleotide	Sarepta Therapeutics
35	Padcev	Enfortumab vedotin-ejfv	12/18/2019	Nectin-4	To treat refractory bladder cancer	Antibody-drug conjugate (ADC)	Seattle Genetics
36	Caplyta	Lumateperone tosylate	12/20/2019	5-HT2A receptor antagonist	To treat schizophrenia	Small molecule	Intra-Cellular Therapies, Inc.
37	Dayvigo	Lemborexant	12/20/2019	Dual antagonist of the orexin OX1 and OX2 receptors	To treat insomnia	Small molecule	Eisai Inc.
38	Enhertu	Fam-trastuzumab deruxtecan-nxki	12/20/2019	Trastuzumab: epidermal growth factor receptor 2 (HER2) deruxtecan: DNA topoisomerase I inhibitor	To treat metastatic breast cancer	Antibody-drug conjugate (ADC)	AstraZeneca and Daiichi Sankyo Company, Limited
39	Ubrelvy	Ubrogepant	12/23/2019	Calcitonin gene-related peptide receptor (CGRP) antagonist	For the acute treatment of migraine with or without aura in adults	Small molecule	Allergan USA, Inc.

Small molecule drugs play an important role in fighting diseases. Although the development of small molecules has slowed slightly in recent years, the 27 small molecule targeted drugs approved in 2019 accounted for nearly 70% of the total number of approved targeted drugs. Small molecule drugs have the advantages of oral bioavailability, pharmacokinetics, drug delivery, production cost, etc., which facilitate the development of this class of drugs, and this comparative advantage will continue in the near future. In addition, small molecules may be used in conjunction with new types of therapies, such as antibody-drug conjugates (ADCs).

In 2019, there was an increase in the number of approved ADCs: polatuzumab vedotin-piiq (Polivy) for relapsed or refractory diffuse large B-cell lymphoma, enfortumab vedotin-ejfv (Padcev) for refractory bladder cancer and fam-trastuzumab deruxtecan-nxki (Enhertu) for metastatic breast cancer. ADCs comprise a monoclonal antibody and cytotoxic agents conjugated via a chemical linker. The specificity of mAbs allows the chemotherapy agents to be selectively delivered to targeted cancer cells, thereby reducing toxicity. Importantly, mAbs such as trastuzumab not only show specificity but also have anticancer effects. To date, seven ADCs have been approved by the FDA for clinical use, and over 100 ADCs are in clinical development.^3^

Two synthetic peptide analogs were approved this year, bremelanotide and afamelanotide. Peptide-based therapy has been applied in various diseases, such as infectious diseases, allergic diseases, autoimmune diseases, sexual dysfunction, and fibrosis. Many efforts have been made to discover novel bioactive peptides. There is much potential for peptide-based therapy.

Another surprising newly emerging field in 2019 was gene therapy. In 2019, two gene therapy products were approved—Givlaari (givosiran) from Alnylam Pharmaceuticals and Vyondys 53 (golodirsen) from Sarepta Therapeutics. Givlaari is an RNA interference (RNAi) therapeutic that targets aminolevulinic acid synthase 1 (ALAS1) to treat acute hepatic porphyria (AHP). This is the second RNAi therapy approved by the FDA; Onpattro (patisiran) was the first. Onpattro was also developed by Alnylam Pharmaceuticals and was approved by the FDA in 2018 to treat hereditary TTR-mediated amyloidosis. Both drugs use enhanced stabilization chemistry (ESC)-GalNAc conjugate technology. Vyondys 53 is an antisense oligonucleotide developed from Sarepta’s phosphorodiamidate morpholino oligomer (PMO) platform; it was approved to treat Duchenne muscular dystrophy (DMD) patients who have a confirmed mutation of the dystrophin gene that causes exon 53 skipping. In the future, more gene therapies currently under development are likely to be approved in the upcoming years, which would bring hope to individuals with severe, life-threatening diseases or rare diseases.

The FDA approved Brukinsa (zanubrutinib) capsules from BeiGene USA, Inc., for the treatment of adult patients with mantle cell lymphoma who received at least one prior therapy. Brukinsa is the first novel anticancer drug developed by a Chinese company and approved by the FDA. It was granted Accelerated Approval, Breakthrough Therapy designation, and Orphan Drug designation. Unfortunately, BeiGene, Ltd., announced that the new drug application (NDA) for zanubrutinib for the treatment of patients with relapsed/refractory mantle cell lymphoma (MCL) was accepted by the National Medical Products Administration (NMPA) on 08/27/2018, but zanubrutinib has not yet been approved.

On 08/30/2018, the China Food and Drug Administration (CFDA) changed its name to the National Medical Products Administration (NMPA), which is administered by the State Administration for Market Regulation (SAMR). In 2019, the NMPA approved 51 new drugs. Herein, we only summarize the eight innovative targeted drugs developed by the Chinese pharmaceutical industry (Table 2), including five small molecules, one antibiotic, one synthetic peptide analog, and two mAbs.

**Table 2:** Targeted drugs developed by Chinese pharmaceutical companies that were approved by the NMPA in 2019

No.	Drug name	Active ingredient	Approval date	Target/Activity	CFDA-approved use on approval date*	Drug class	Company
1	Bite	Carrimycin	6/24/19	β-lactamase	Bacterial infection of the upper respiratory tract, resistant *Mycobacterium tuberculosis* infection	Small molecule	Shengyang Tonglian Group Co., Ltd.
2	Fu Laimei Polyethylene Glycol Loxenatide Injection	PEG-loxenatide	5/7/19	GLP-1 receptor antagonist	Type 2 diabetes	Synthetic peptide analog	Hansoh Pharma
3		Camrelizumab	5/30/19	PD-1 inhibitor	Recurrent or refractory classical Hodgkin lymphoma	Monoclonal antibody (mAb)	Jiangsu Hengrui Medicine Co., Ltd.
4	Xinbike	Benvitimod	5/31/19	Aryl hydrocarbon receptor (AhR) agonist	Psoriasis	Small molecule	Tianji Pharma
5	GV-971	Sodium oligomannate	11/2/19	gut microbiota	Mild to moderate Alzheimer’s disease	Small molecule	Green Valley
6		Flumatinib mesylate	11/26/19	BCR-Abl inhibitor	Chronic myelogenous leukemia	Small molecule	Hansoh Pharma
7	ZEJULA	Niraparib tosylate	12/27/19	PARP1/2 inhibitor	Recurrent epithelial ovarian cancer	Small molecule	Zai Lab
8		Tislelizumab	12/27/19	PD-1 inhibitor	Classical Hodgkin lymphoma (cHL)	Monoclonal antibody (mAb)	BeiGene
9		Remimazolam tosylate	12/27/19	GABA_A_ receptor antagonist	Sedation for routine gastroscopy	Small molecule	Jiangsu Hengrui Medicine Co. Ltd.

Two cancer immunotherapy drugs that target PD-1 have been approved, camrelizumab from Jiangsu Hengrui Medicine Co. and tislelizumab from BeiGene. Camrelizumab and tislelizumab are humanized IgG4 anti-PD-1 monoclonal antibodies that block the binding of PD-1 to its ligands. The first PD-1 inhibitor to hit the market was pembrolizumab (Keytruda), which was approved by the FDA in 2014. Since, ten PD-1/PD-L1 cancer immunotherapy drugs have come on the market worldwide, four of which were developed by Chinese pharmaceutical companies; these drugs are camrelizumab, tislelizumab, and sintilimab developed by Innovent Biologics and Eli Lilly and toripalimab developed by Shanghai Junshi Bioscience Co., Ltd. Following the FDA approval of Brukinsa (zanubrutinib), tislelizumab was the first drug developed by BeiGene to be approved in China. These young Chinese pharmaceutical companies show great potential in drug discovery, especially for novel targets. As China is the world’s second-largest pharmaceutical market, we expect to see an increase in the number of novel drugs developed in China.

New classes of drugs developed using new technologies provide new treatment options and hope to patients with fatal diseases. For example, the cancer mortality rate in the US declined by 29% from 1991 to 2017. This success is partially due to targeted therapies such as the BRAF inhibitor Zelboraf (vemurafenib) and the anti-CTLA4 antibody Yervoy (ipilimumab).^4^ In addition, emerging innovative therapeutic approaches such as CAR-T cell therapy for cancer and CRISPR-Cas9 gene editing could potentially make a difference for patients.

In 2020, Signal Transduction and Targeted Therapy aims to be the leading forum for research addressing unmet medical needs, including cancer, immune disorders, infectious diseases, virus such as SARS-CoV-2, diabetes, cardiovascular diseases, inflammation, central nervous system diseases, and other pathologies. We expect to publish papers on the discovery and development of new targets and therapeutic options that may have a significant impact on healthcare in the future.
